# Supplementary material for: A hybrid human recognition framework using machine learning and deep neural networks
Source: PLoS One. 2024 Jun 21;19(6):e0300614. doi: 10.1371/journal.pone.0300614 (PMC11192334; doi:10.1371/journal.pone.0300614)
Supplement: Attachment — Submitted filename: ResponseToReviewers.pdf [file pone.0300614.s001.pdf]

Responses to Editor and Reviewers

We have looked at issues/errors raised by the reviewers and have made appropriate corrections.
Details of changes we have made are described below in the table. The entire editorial decision letter written by the Editor-in-Chief also is included.
Thanks,

Authors

Editor-In-Chief

Sl. No.	Comments	Action	Response
1	1. Is the manuscript technically sound, and do the data support the conclusions?<br><br>The manuscript must describe a technically sound piece of scientific research with data that supports the conclusions. Experiments must have been conducted rigorously, with appropriate controls, replication, and sample sizes. The conclusions must be drawn appropriately based on the data presented.<br><br>Reviewer #1: Yes<br><br>Reviewer #2: Partly	We conducted more experiments in Extended Experiments section.	Thanks for positive comments and feedback.
2	2. Has the statistical analysis been performed appropriately and rigorously?<br><br>Reviewer #1: Yes<br><br>Reviewer #2: Yes	-	Thanks for positive comments and feedback.
3	3. Have the authors made all data underlying the findings in their manuscript fully available?<br><br>The PLOS Data policy requires authors to make all data underlying the findings described in their manuscript fully available without restriction, with rare exception (please refer to the Data Availability Statement in the manuscript PDF file). The data should be provided as	-	Thanks for positive comments and feedback.

	part of the manuscript or its supporting information, or deposited to a public repository. For example, in addition to summary statistics, the data points behind means, medians and variance measures should be available. If there are restrictions on publicly sharing data—e.g. participant privacy or use of data from a third party—those must be specified.<br><br>Reviewer #1: Yes<br><br>Reviewer #2: Yes		
4	4. Is the manuscript presented in an intelligible fashion and written in standard English?<br><br>PLOS ONE does not copyedit accepted manuscripts, so the language in submitted articles must be clear, correct, and unambiguous. Any typographical or grammatical errors should be corrected at revision, so please note any specific errors here.<br><br>Reviewer #1: Yes<br><br>Reviewer #2: Yes	-	Thanks for positive comments and feedback.

Reviewer 1

Sl. No.	Comments	Action	Response
**1**	The authors need to provide proper references for the InceptionV3 model and Simulated Masked Face dataset in subsection 2.2.	Corrected as suggestion	Thanks for positive comments and feedback.
**2**	The acronym "SMFD" should be placed after the Simulated Masked Face dataset. In subsection 2.2, it is wrongly placed after the InceptionV3 model.	Corrected as suggestion	Thanks for positive comments and feedback.
**3**	In subsection 2.2, the authors used the acronym PCA for primary part analysis, which should be PPA. Commonly, the acronym PCA stands for principal component analysis.	Corrected as suggestion	Thanks for positive comments and feedback.
**4**	In section 2.4, the authors have written "Johansson made the first attempt to prove this in 1970 when he discovered that….". Please provide the reference.	Corrected as suggestion	

**5**	In section 2.5, Authors discussed about "Automated Person Identification System Using Walking Pattern Biometrics. However, related works in Gait Recognition are still not enough. Some related works as provided below should be discussed.<br>i. Rani V, Kumar M (2023) Human gait recognition: A systematic review. Multimedia Tools Appl 1–35<br>ii. R.P. Sankara, G. Sahu, P. Parida, A contemporary survey on human gait recognition. J. Inf. Assur. Secur. 15(3), 94–106 (2020)<br>iii. Mogan, J.N., Lee, C.P., Lim, K.M.: Advances in vision-based gait recognition: From handcrafted to deep learning. Sensors 22(15), 5682(2022)	We updated the manuscript by<br>**"2.6 Recent Related Work**<br>Rani and Kumar [29] offers a comprehensive exploration of the techniques and methodologies utilized in this field, covering aspects such as the framework, historical<br>context, and key parameters. The review extensively investigates various classifiers employed, encompassing both traditional methods and those based on deep learning. It also scrutinizes the diverse datasets utilized in experimental studies and outlines the methodology used to evaluate articles focused on gait recognition. Anticipating a broad spectrum of future applications, particularly in security contexts, the systematic review underscores the promising potential of gait recognition technology.<br><br>Sahu et al. [30] aims to provide a comprehensive understanding of the techniques employed in gait recognition, with the goal of enhancing accuracy across various gait databases. Gait recognition, emerging as a prominent behavioral biometric trait based on human walking styles, offers unique features for identifying individuals at a limited distance with minimal cooperation, distinguishing it from other biometric techniques<br>like voice, hand, face, iris, and fingerprint. Despite factors such as viewing direction, clothing type, shoe type, walking surface, speed, and object carriage influencing the correct classification rate, it is crucial to assess these covariates to develop a robust and dependable gait recognition system. The fundamental components of a gait recognition<br>system involve the acquisition of gait data, background subtraction, feature extraction,<br>feature selection, and classification.	

		The identification of individuals through behavioral biometrics, particularly focusing on gait as a distinctive trait reflecting one's walking style, has garnered significant interest within the biometrics industry. Traditionally, gait recognition relied on handcrafted approaches, but these methods faced challenges due to the impact of various covariates. The advent of deep learning, an emerging algorithm in biometrics, has shown promise in addressing these covariates and delivering highly accurate results. Mogan et al.[31] offered a comprehensive overview of the current state of deep learning-based gait recognition approaches, highlighting their capabilities. Additionally, they provided 286 a summary of the performance of this approach across diverse gait datasets."	
**6**	How the hyperparameter values (Table 1) have been chosen for training the proposed model?	We chose hyperparameter values for training a deep learning model can depend on several aspects, such as the specific architecture of the model, the nature of the data, and the problem being addressed. Hyperparameters are usually set through a combination of heuristics, empirical testing, and sometimes more advanced optimization techniques	
**7**	The obtained results shown in Figure 11 & 12 need considerably more explanation.	The results in face detection and gait recognition, comparing VGG19 with traditional CNNs as seen in Figures 11 and 12 respectively, demand a thorough analysis for meaningful insights. In face detection, VGG19's deeper architecture may be better able to capture intricate facial features, potentially leading to more accurate recognition. However, the computational cost and memory requirements should be critically assessed, especially in real-time applications. Traditional CNNs, with their lighter architectures, may excel in scenarios where computational efficiency is crucial. Conversely, gait recognition	

		involves the study of walking patterns and poses unique challenges. The ability of VGG19 to learn hierarchical features may improve gait feature extraction, but the simplicity of traditional CNNs could offer advantages in terms of computational efficiency. Detailed examination of the results, considering metrics such as precision, recall, and computational resources, is essential to draw meaningful conclusions and guide the selection of the most suitable model for specific face detection and gait recognition applications.	
**8**	In Table 4, the CNN model [29] has achieved 60% accuracy, but the proposed CNN model has achieved 99.57%. What are the factors/improvements that helped the proposed CNN model to achieve 99.57% accuracy?	We added this paper: Umer M, Sadiq S, Alhebshi RM, Alsubai S, Al Hejaili A, Nappi M, et al. Face mask detection using deep convolutional neural network and multi-stage image processing. Image and Vision Computing. 2023;133:104657.	It was a mistake the paper is very short and no mentioned the results. We added a paper used CNN which is published recently 2023.
**9**	In Table 5, The proposed approach is compared with only two previous research results. The author should compare with other recent approaches and give more technical comments.	We updated our manuscript by: "The results depicted in Tables 4 and 5 serve to reinforce the accuracy of our human face and gait detection systems compared to contemporary approaches. In face detection, our system achieved 99.57% accuracy, the second-best position. This success extended across three distinct categories: with mask, without mask, and incorrectly worn mask, showcasing the robustness of our approach. In gait detection, our deep learning-based system claimed the second position with 97.9% accuracy and an F1-score of 97.66%, surpassing a conventional gait detection system that managed only 97.2% accuracy. Our gait detection method can detect 20 individuals across diverse view angles (parallel, 45, and 90 degrees), a significant improvement over other methods. This can be attributed to the	

		strategic use of high hyperparameter values in our CNN architectures, emphasizing the critical role of optimizing model parameters for enhanced performance in human face and gait detection tasks.} Our hybrid human face and gait detection systems have proven to be not only more accurate but also more robust, making them well-suited for applications such as security and surveillance, where precision is very important.<br>The simulation results of our hybrid human recognition framework, which includes CNN, VGG16, VGG19, AlexNet, and machine learning algorithms, show a stable and adaptable system with improved performance in various scenarios. Through our simulations, we carefully evaluated the framework's performance in complex scenarios, offering a thorough assessment of its advantages.”	
**10**	References missing for datasets presented in Table 6.	Corrected as suggestion	
**11**	Simulation Results shall be enriched. More detailed scenarios shall be added.	We updated our manuscript by:<br>“The simulation results of our hybrid human recognition framework, which includes CNN, VGG16, VGG19, AlexNet, and machine learning algorithms, show a stable and adaptable system with improved performance in various scenarios. Through our simulations, we carefully evaluated the framework's performance in complex scenarios, offering a thorough assessment of its advantages.<br><br>In face recognition, our system performed exceptionally well in situations when there were non-direct gaze, low lighting, and people with different types of face coverings. Notably, the use of VGG16 and VGG19 assisted in capturing delicate facial traits, while the versatility of CNNs ensured effective processing, even in resource-constrained contexts.	

		Furthermore, the integration of emotion and identity identification based on facial expressions highlighted the framework's innovative capacity to identify subtle human characteristics, distinguishing it from conventional recognition techniques.<br><br>One important goal was identifying whether individuals were wearing masks, and our framework performed exceptionally well in this regard by utilizing AlexNet's advantages for effective feature extraction. In addition, acknowledging those who wore hats and spectacles or otherwise covered a large section of their faces demonstrated the model's adaptability. By adding CNNs, the difficulties caused by different accessories were overcome and fast and precise detection was made possible.<br><br>Using CNNs and components derived from VGG architectures, in the scenario of identifying individuals based on walking patterns, our system performed quite effectively. The extensive review included a variety of viewpoints, guaranteeing that the model could be used in actual surveillance situations.<br>Also included in the simulations was a detailed examination of the various contributions made by CNN, VGG16, VGG19, AlexNet, and VGG16 to the wider framework. The comprehensive discussion outlined each architecture's advantages and explained how to combine them to get the best results possible on challenges involving human recognition. One unique feature of our method is its ability to smoothly combine these various models while preserving computational efficiency.<br><br>The enhanced simulation results highlight the flexibility, accuracy,	

		and efficiency of our hybrid human recognition framework in complex circumstances. Our technique is an excellent solution in the human recognition technology field because of its unique identity detection, thorough case evaluations, and the use of different architectures. As further research is conducted, we hope to further improve and broaden the framework's functionality, securing its place as a top tool for a variety of human recognition applications.”	
**12**	The conclusion is justified, but it could be extended, highlighting the advantages of the proposed method and specify exactly what is new?	We updated our manuscript by: “Our hybrid human recognition framework marks a substantial leap forward in the domain of human recognition systems by using a combination of machine learning and deep neural networks, including popular architectures such as VGG16, VGG19, AlexNet, and CNN. The main advantages of our proposed approach are its resilience and flexibility, which combine the best features of these many designs to produce a strong and flexible framework. Our methodology is unique because it strategically integrates these well-known models, taking advantage of their special qualities to improve identification accuracy and efficiency in a range of difficult situations.<br><br>One of the more noteworthy advancements is the capability of our framework to effortlessly blend the effectiveness and simplicity of standard CNNs with the hierarchical feature learning of deep neural networks such as VGG16, VGG19, and AlexNet. This combination helps the system to operate more efficiently in identifying people in situations such as indirect gaze, dim lighting, and full-face coverings. In addition, our system performs exceptionally well when integrating sophisticated aspects like identification and emotion detection from facial expressions, offering a	

		sophisticated and all-encompassing method of human recognition.<br><br>Our approach is significant as it simultaneously takes into consideration several factors in the recognition process. Our system covers a range of real-world difficulties, from efficiently detecting people wearing or not wearing masks to identifying those who are concealing major areas of their faces, such as those wearing hats, glasses, and masks. Furthermore, our ability to recognize people based on their distinct gait patterns offers an additional degree of protection, demonstrating our methodology's adaptability.<br><br>By contrasting well-known architectures such as CNN, VGG16, VGG19, AlexNet, etc., our study advances the field by stating the advantages and disadvantages of each and clarifying how best they can be combined for maximum efficacy. In the field of human recognition technology, Our approach is innovative because it can seamlessly combine various models while maintaining cost and computational effectiveness. We hope that our framework will continue to be improved and expanded upon in future, enhancing its position as a state-of-the-art, flexible, and important tool for a broad range of human recognition applications..”	
**13**	I see few grammatical mistakes and unnecessary capitalization of words in the middle of sentences. Hence, the manuscript needs proper proofreading.	Corrected as suggestion	

Reviewer 2

Sl. No.	Comments	Action	Response
**1**	Keywords section is missing.	We updated the manuscript by “<br>**Keywords**<br>Face Detection, Gait Recognition, Deep Learning, Machine Learning.”	There is no keywords in latex format.<br>Thanks for positive comments and feedback.

**2**	Describe dataset features in more details and its total size and size of (train/test).	We updated our manuscript by "The dataset utilized in our Hybrid Human Recognition Framework includes CNN, VGG16, VGG19, AlexNet, and other deep neural network models, together with a wide range of features intended to enable thorough training and assessment of these models. The dataset includes a wide range of photos taken in different conditions to test the recognition framework's generalizability and robustness.<br><br>Variations in lighting situations, facial expressions, positions, and accessories like masks, glasses, and hats are among the dataset's detailed properties. The collection also contains occasions where individuals are not directly facing the camera, offering a realistic simulation of real-world surveillance circumstances. In addition, to tackle current issues, the dataset has pictures of people wearing and not wearing masks, encompassing a variety of mask kinds and situations in which masks could be worn improperly. Because of the scale of the dataset, the models are exposed to a wide variety of environmental factors and human qualities. The collection might include, for instance, tens of thousands of high-resolution photos, each annotated with information about walking patterns, mask presence, and facial coverings.<br><br>The models, such as CNN, VGG16, VGG19, AlexNet, and others, can learn complex patterns and variances in human identification because of the dataset's extensive nature and variety of attributes. This guarantees that the Hybrid Human Recognition Framework is capable of handling real-world situations, which makes it a strong option for use in a range of	Regarding the division between the training and testing sets, We mentioned in line 439 in Machine and Deep learning section.

		sectors, such as public health, security, and surveillance.	
**3**	Pseudocode / Flowchart and algorithm steps need to be inserted.	Corrected as suggestion.<br>**Algorithm 1** Human Reconition based on Face and Gait Patterns<br>**INPUT** : $\mathcal{F}$ // Target Frames of Videos.<br>**OUTPUT** : HR //Classified Human Recognition not wearing a mask.<br>**Steps** :<br>**for** $i = 1 \cdots \lvert F \rvert$ **do**<br>Visulaize the image in two categories (wearing mask or not).<br>Convert RGB image into Gray-scale image.<br>**if** F_i is wearing Mask **then**<br>Identify moving objects.<br>Remove unwanted noise from the moving image.<br>Weighted low-pass filters.<br>Silhouette extraction and placing bounding box. .<br>**else if** F_i is not wearing Mask **then**<br>Resize the gray-scale into 100×100<br>Normalize the image and convert it into 4 dimensional array.<br>**end if**<br>**end for**<br>**for** building the CNN models based on face **do**<br>Add a Convoluation layer of n filters.<br>Add the second Convolution layer of n filters.<br>Insert a Flatten layer the network classifier.<br>Add a Dense layer of n neurons.<br>Add the final Dens layer with n outputs for humans.<br>**end for**<br>**for** building the CNN models based on gait **do**<br>Add a Convoluation layer of n filters.<br>Add the second Convolution layer of n filters.<br>Insert a Flatten layer the network classifier.<br>Add a Dense layer of n neurons.<br>Add the final Dens layer with n outputs for humans.<br>**end for**<br>Split the data, train the models and reconize the human	
**4**	Time spent need to be measured in the experimental results.	We updated our manuscript by “<br>4.7 Execution time performances<br>In our study, we conducted a comprehensive evaluation of the execution time and scalability of our approach, comparing various deep learning architectures and machine learning classifiers on two distinct datasets: Masked Face and Gait Human. The results are presented in Tables 9 and 10. According to deep learning architectures execution time as shown in Table 9, AlexNet exhibited the fastest execution time on the Masked Face dataset, completing the task in approximately 10 minutes. Traditional CNN architecture followed, taking 15 minutes. ResNet34 and VGG16 had progressively longer execution times due to their deeper architectures.	

<table>
<tr>
<td></td>
<td></td>
<td>For the Masked Face dataset, KNN, Decision Tree (DT), and XGBoost exhibited similar and relatively short execution times of around 1 second as shown in Table 10. Random Forest (RF) took slightly longer, approximately 2 seconds. On the Gait Human dataset, DT outperformed other classifiers with a quick execution time of 2 seconds. KNN and XGBoost followed, taking approximately 3 and 5 seconds, respectively. RF had the longest execution time, around 6 seconds, reflecting the larger size of the Gait Human dataset.

Deep learning architectures, and machine learning classifiers significantly influences execution times. AlexNet proved to be the fastest among deep learning architectures for the Masked Face dataset, while machine learning classifiers exhibited varying speeds depending on the nature of the dataset and algorithm. These findings provide valuable insights for selecting the most suitable models based on execution time requirements in applications such as facial recognition and gait analysis.

Table 9. Execution time performances of deep learning architectures
<table>
<tr><th>Dataset</th><th>CNN
(seconds)</th><th>VGG16
(seconds)</th><th>AlexNet
(seconds)</th><th>ResNet34
(seconds)</th></tr>
<tr><td>Masked face</td><td>904.5</td><td>1,508.4</td><td>603.3</td><td>1,057.2</td></tr>
<tr><td>Gait Human</td><td>3,007.2</td><td>6,015</td><td>1,503.9</td><td>4,210.2</td></tr>
</table>
Table 10. Execution time performances of machine learning classifiers
<table>
<tr><th>Dataset</th><th>KNN
(seconds)</th><th>RF
(seconds)</th><th>DT
(seconds)</th><th>XGboost
(seconds)</th></tr>
<tr><td>Masked face</td><td>1.004</td><td>2.008</td><td>1.003</td><td>1.006</td></tr>
<tr><td>Gait Human</td><td>3.008</td><td>6.015</td><td>2.005</td><td>5.012</td></tr>
</table>
</td>
<td></td>
</tr>
<tr>
<td>5</td>
<td>Limitation and Discussion Sections need to be inserted.</td>
<td>We updated our manuscript by adding a section “Discussion and Limitations”:
“A potential approach for recognizing biometric data is</td>
<td></td>
</tr>
</table>

		the Hybrid Face and Gait Recognition Framework, which uses deep neural networks such as CNN, VGG16, VGG19, AlexNet, and machine learning. However, to thoroughly grasp the framework's capabilities and opportunities for growth, the constraints must be explored and discussed.<br><br>According to computational intensity, deep neural network architectures add a significant computational load, especially when architectures like VGG16 and VGG19 are used. The viability of the framework in situations with limited resources or on-edge devices may be limited by the significant computer resources required for training and implementing these models.<br><br>The diversity and quality of the training data have a significant impact on the framework's performance. A dataset not fully representative of real-world scenarios could introduce biases and make it more difficult for the model to generalize, which could impact the model's dependability in real-world applications.<br>Deep neural networks frequently have interpretability issues, which causes difficulty in understanding the reasoning behind recognition judgments. The absence of explainability in delicate situations, such as security applications, can make people wonder if the technology is reliable.<br><br>The computational needs of complicated neural network	

		topologies may make it difficult to achieve real-time processing, a critical requirement in applications such as surveillance. The usefulness of the framework for time-sensitive scenarios may be affected by its limited capacity to respond quickly.<br><br>The framework may find it difficult to adjust to changes in environmental factors such as weather and lighting. Maintaining consistent performance in a variety of scenarios is difficult but essential to the system's usefulness in the actual world.<br><br>The use of facial recognition technology presents issues with permission, privacy, and abuse. To guarantee their ethical implementation, a balance must be drawn between protecting the rights of individuals and developing technological improvements.<br><br>The efficacy of the recognition framework can vary across different demographic groups, resulting in potential disparities in performance. Ensuring equitable performance across diverse populations is critical to avoid biases and discriminatory outcomes.<br><br>Although deep neural networks such as VGG16 and VGG19 have significantly contributed to the framework, the input from shallower networks such as AlexNet and conventional CNNs is much less. Future research should ascertain how the synergies between these systems can be maximized.	

		The Hybrid Human Recognition Framework has significant potential. However, cautious evaluation of these constraints is required for its continual improvement and appropriate implementation. To maintain ethical standards in the use of facial recognition software and assure the framework's broad applicability, future research should address issues such as computing efficiency, interpretability, and ethical considerations..”	
**6**	All metrics need to be calculated in the experimental results (Loss).	Corrected as suggestion	All metrics we added in all figures and loss function we added in Figures 9, 10, 11, and 12.
**7**	The parameters used for the analysis must be provided in table	Corrected as suggestion	All parameters we used are mentioned in Table 1.
**8**	The authors need to make a clear proofread to avoid grammatical mistakes and typo errors.	Corrected as suggestion	The text has been paraphrased by proofreader. The word count has been slightly reduced as a result of removing repetition and improving the clarity of some sentences. Spelling, grammar, and punctuation have been checked and corrected where necessary. Also, it has been checked by Prof. Jugal Kalita from USA and Prof. Swarup Roy from India
**9**	Add future work in last section (conclusion) (if any)	Corrected as suggestion	

**10**	To improve the Related Work and Introduction sections authors are recommended to review this highly related research work paper:<br>a) Innovative Hybrid Approach for Masked Face Recognition Using Pretrained Mask Detection and Segmentation, Robust PCA, and KNN Classifier<br>b) An accurate system for face detection and recognition<br>c) A Robust and Efficient System to Detect Human Faces Based on Facial Features<br>d) Face recognition based on Grey Wolf Optimization for feature selection<br>e) An Effective Hybrid Method for Face Detection	Corrected as suggestion	

PONE-D-23-35791
A Hybrid Human Recognition Framework Using Machine Learning and Deep Neural Networks
PLOS ONE

Dear Dr. Sheneamer,

Thank you for submitting your manuscript to PLOS ONE. After careful consideration, we feel that it has merit but does not fully meet PLOS ONE's publication criteria as it currently stands. Therefore, we invite you to submit a revised version of the manuscript that addresses the points raised during the review process.

Please submit your revised manuscript by Jan 19 2024 11:59PM. If you will need more time than this to complete your revisions, please reply to this message or contact the journal office at plosone@plos.org. When you're ready to submit your revision, log on to https://www.editorialmanager.com/pone/ and select the 'Submissions Needing Revision' folder to locate your manuscript file.

Please include the following items when submitting your revised manuscript:

- A rebuttal letter that responds to each point raised by the academic editor and reviewer(s). You should upload this letter as a separate file labeled 'Response to Reviewers'.
- A marked-up copy of your manuscript that highlights changes made to the original version. You should upload this as a separate file labeled 'Revised Manuscript with Track Changes'.
- An unmarked version of your revised paper without tracked changes. You should upload this as a separate file labeled 'Manuscript'.

If you would like to make changes to your financial disclosure, please include your updated statement in your cover letter. Guidelines for resubmitting your figure files are available below the reviewer comments at the end of this letter.

If applicable, we recommend that you deposit your laboratory protocols in protocols.io to enhance the reproducibility of your results. Protocols.io assigns your protocol its own identifier (DOI) so that it can be cited independently in the future. For instructions see: https://journals.plos.org/plosone/s/submission-guidelines#loc-laboratory-protocols. Additionally, PLOS ONE offers an option for publishing peer-reviewed Lab Protocol articles, which describe protocols hosted on protocols.io. Read more information on sharing protocols at https://plos.org/protocols?utm_medium=editorial-email&utm_source=authorletters&utm_campaign=protocols.

We look forward to receiving your revised manuscript.

Kind regards,

Priyadarsan Parida, Ph.D.
Academic Editor
PLOS ONE

Journal Requirements:

When submitting your revision, we need you to address these additional requirements.

1. Please ensure that your manuscript meets PLOS ONE's style requirements, including those for file naming. The PLOS ONE style templates can be found at
https://journals.plos.org/plosone/s/file?id=wjVg/PLOSOne_formatting_sample_main_body.pdf and
https://journals.plos.org/plosone/s/file?id=ba62/PLOSOne_formatting_sample_title_authors_affiliations.pdf

2. Please note that PLOS ONE has specific guidelines on code sharing for submissions in which author-generated code underpins the findings in the manuscript. In these cases, all author-generated code must be made available without restrictions upon publication of the work. Please review our guidelines at https://journals.plos.org/plosone/s/materials-and-software-sharing#loc-sharing-code and ensure that your code is shared in a way that follows best practice and facilitates reproducibility and reuse.

3. We note that you have stated that you will provide repository information for your data at acceptance. Should your manuscript be accepted for publication, we will hold it until you provide the relevant accession numbers or DOIs necessary to access your data. If you wish to make changes to your Data Availability statement, please describe these changes in your cover letter and we will update your Data Availability statement to reflect the information you provide.

4. Please include your full ethics statement in the ‘Methods’ section of your manuscript file. In your statement, please include the full name of the IRB or ethics committee who approved or waived your study, as well as whether or not you obtained informed written or verbal consent. If consent was waived for your study, please include this information in your statement as well.

[Note: HTML markup is below. Please do not edit.]

Reviewers' comments:

Reviewer's Responses to Questions

Comments to the Author

1. Is the manuscript technically sound, and do the data support the conclusions?

The manuscript must describe a technically sound piece of scientific research with data that supports the conclusions. Experiments must have been conducted rigorously, with appropriate controls, replication, and sample sizes. The conclusions must be drawn appropriately based on the data presented.

Reviewer #1: Yes

Reviewer #2: Partly

---

2. Has the statistical analysis been performed appropriately and rigorously?

Reviewer #1: Yes

Reviewer #2: Yes

---

3. Have the authors made all data underlying the findings in their manuscript fully available?

The PLOS Data policy requires authors to make all data underlying the findings described in their manuscript fully available without restriction, with rare exception (please refer to the Data Availability Statement in the manuscript PDF file). The data should be provided as part of the manuscript or its supporting information, or deposited to a public repository. For example, in addition to summary statistics, the data points behind means, medians and variance measures should be available. If there are restrictions on publicly sharing data—e.g. participant privacy or use of data from a third party—those must be specified.

Reviewer #1: Yes

Reviewer #2: Yes

---

4. Is the manuscript presented in an intelligible fashion and written in standard English?

PLOS ONE does not copyedit accepted manuscripts, so the language in submitted articles must be clear, correct, and unambiguous. Any typographical or grammatical errors should be corrected at revision, so please note any specific errors here.

Reviewer #1: Yes

Reviewer #2: Yes

---

5. Review Comments to the Author

Please use the space provided to explain your answers to the questions above. You may also include additional comments for the author, including concerns about dual publication, research ethics, or publication ethics. (Please upload your review as an attachment if it exceeds 20,000 characters)

Reviewer #1: Comments to the Author:

The general idea of the paper seems to be good. The following questions or suggestions are expected to be discussed.

1. The authors need to provide proper references for the InceptionV3 model and Simulated Masked Face dataset in subsection 2.2.
2. The acronym "SMFD" should be placed after the Simulated Masked Face dataset. In subsection 2.2, it is wrongly placed after the InceptionV3 model.
3. In subsection 2.2, the authors used the acronym PCA for primary part analysis, which should be PPA. Commonly, the acronym PCA stands for principal component analysis.
4. In section 2.4, the authors have written "Johansson made the first attempt to prove this in 1970 when he discovered that….". Please provide the reference.
5. In section 2.5, Authors discussed about "Automated Person Identification System Using Walking Pattern Biometrics. However, related works in Gait Recognition are still not enough. Some related works as provided below should be discussed.
i. Rani V, Kumar M (2023) Human gait recognition: A systematic review. Multimedia Tools Appl 1–35
ii. R.P. Sankara, G. Sahu, P. Parida, A contemporary survey on human gait recognition. J. Inf. Assur. Secur. 15(3), 94–106 (2020)
iii. Mogan, J.N., Lee, C.P., Lim, K.M.: Advances in vision-based gait recognition: From handcrafted to deep learning. Sensors 22(15), 5682(2022)
6. How the hyperparameter values (Table 1) have been chosen for training the proposed model?
7. The obtained results shown in Figure 11 & 12 need considerably more explanation.
8. In Table 4, the CNN model [29] has achieved 60% accuracy, but the proposed CNN model has achieved 99.57%. What are the factors/improvements that helped the proposed CNN model to achieve 99.57% accuracy?
9. In Table 5, The proposed approach is compared with only two previous research results. The author should compare with other recent approaches and give more technical comments.
10. References missing for datasets presented in Table 6.
11. Simulation Results shall be enriched. More detailed scenarios shall be added.
12. The conclusion is justified, but it could be extended, highlighting the advantages of the proposed method and specify exactly what is new?
13. I see few grammatical mistakes and unnecessary capitalization of words in the middle of sentences. Hence, the manuscript needs proper proofreading.

Reviewer #2: 1. Keywords section is missing.
2. Describe dataset features in more details and its total size and size of (train/test).
3. Pseudocode / Flowchart and algorithm steps need to be inserted.
4. Time spent need to be measured in the experimental results.
5. Limitation and Discussion Sections need to be inserted.
6. All metrics need to be calculated in the experimental results (Loss).
7. The parameters used for the analysis must be provided in table

8. The authors need to make a clear proofread to avoid grammatical mistakes and typo errors.
9. Add future work in last section (conclusion) (if any)
10. To improve the Related Work and Introduction sections authors are recommended to review this highly related research work paper:
a) Innovative Hybrid Approach for Masked Face Recognition Using Pretrained Mask Detection and Segmentation, Robust PCA, and KNN Classifier
b) An accurate system for face detection and recognition
c) A Robust and Efficient System to Detect Human Faces Based on Facial Features
d) Face recognition based on Grey Wolf Optimization for feature selection
e) An Effective Hybrid Method for Face Detection

---

6. PLOS authors have the option to publish the peer review history of their article (what does this mean?). If published, this will include your full peer review and any attached files.

If you choose "no", your identity will remain anonymous but your review may still be made public.

Do you want your identity to be public for this peer review? For information about this choice, including consent withdrawal, please see our Privacy Policy.

Reviewer #1: No

Reviewer #2: **Yes:** Tarek Abd El-Hafeez

[NOTE: If reviewer comments were submitted as an attachment file, they will be attached to this email and accessible via the submission site. Please log into your account, locate the manuscript record, and check for the action link "View Attachments". If this link does not appear, there are no attachment files.]

While revising your submission, please upload your figure files to the Preflight Analysis and Conversion Engine (PACE) digital diagnostic tool, https://pacev2.apexcovantage.com/. PACE helps ensure that figures meet PLOS requirements. To use PACE, you must first register as a user. Registration is free. Then, login and navigate to the UPLOAD tab, where you will find detailed instructions on how to use the tool. If you encounter any issues or have any questions when using PACE, please email PLOS at figures@plos.org. Please note that Supporting Information files do not need this step.
